# Evaluation of the Murine Immune Response to *Xenopsylla cheopis* Flea Saliva and Its Effect on Transmission of *Yersinia pestis*

**DOI:** 10.1371/journal.pntd.0003196

**Published:** 2014-09-25

**Authors:** Christopher F. Bosio, Austin K. Viall, Clayton O. Jarrett, Donald Gardner, Michael P. Rood, B. Joseph Hinnebusch

**Affiliations:** 1 Laboratory of Zoonotic Pathogens, Rocky Mountain Laboratories, National Institute of Allergy and Infectious Diseases, National Institutes of Health, Hamilton, Montana; 2 Veterinary Pathology Section, Rocky Mountain Laboratories, National Institute of Allergy and Infectious Diseases, National Institutes of Health, Hamilton, Montana; 3 Los Angeles County Department of Public Health, Baldwin Park, California; University of Tennessee, United States of America

## Abstract

**Background/Aims:**

Arthropod-borne pathogens are transmitted into a unique intradermal microenvironment that includes the saliva of their vectors. Immunomodulatory factors in the saliva can enhance infectivity; however, in some cases the immune response that develops to saliva from prior uninfected bites can inhibit infectivity. Most rodent reservoirs of *Yersinia pestis* experience fleabites regularly, but the effect this has on the dynamics of flea-borne transmission of plague has never been investigated. We examined the innate and acquired immune response of mice to bites of *Xenopsylla cheopis* and its effects on *Y. pestis* transmission and disease progression in both naïve mice and mice chronically exposed to flea bites.

**Methods/Principal Findings:**

The immune response of C57BL/6 mice to uninfected flea bites was characterized by flow cytometry, histology, and antibody detection methods. In naïve mice, flea bites induced mild inflammation with limited recruitment of neutrophils and macrophages to the bite site. Infectivity and host response in naïve mice exposed to flea bites followed immediately by intradermal injection of *Y. pestis* did not differ from that of mice infected with *Y. pestis* without prior flea feeding. With prolonged exposure, an IgG1 antibody response primarily directed to the predominant component of flea saliva, a family of 36–45 kDa phosphatase-like proteins, occurred in both laboratory mice and wild rats naturally exposed to *X. cheopis*, but a hypersensitivity response never developed. The incidence and progression of terminal plague following challenge by infective blocked fleas were equivalent in naïve mice and mice sensitized to flea saliva by repeated exposure to flea bites over a 10-week period.

**Conclusions:**

Unlike what is observed with many other blood-feeding arthropods, the murine immune response to *X. cheopis* saliva is mild and continued exposure to flea bites leads more to tolerance than to hypersensitivity. The immune response to flea saliva had no detectable effect on *Y. pestis* transmission or plague pathogenesis in mice.

## Author Summary

The saliva of blood-feeding arthropods contains a variety of components that prevent blood clotting and interfere with the immune system of the vertebrate host. These properties have been shown to enhance or inhibit the transmission of different pathogens transmitted by arthropods. *Yersinia pestis*, the bacterial agent of plague, is maintained in nature by flea to rodent transmission cycles. Most rodents live in close association with fleas and are constantly being bitten by them, but the influence this has on plague transmission is unknown - previous studies used laboratory animals which have never experienced a flea bite. We found that flea bites caused a mild inflammatory response in mice, and eventually an antibody response to components of flea saliva, but did not significantly affect pathogenesis. The transmission of *Y. pestis* by infected fleas and the incidence rate of bubonic plague mortality were the same in mice that had been exposed to frequent uninfected flea bites and mice with no prior exposure to fleas. Therefore, in contrast to what has been shown for many other arthropod-borne disease systems, vector saliva did not enhance or inhibit *Y. pestis* infection in mice, regardless of the immune status of the host to flea saliva.

## Introduction

*Yersinia pestis*, the etiologic agent of plague, is adapted to flea-borne transmission and is a highly invasive, virulent pathogen. Infected fleas typically transmit small numbers of *Y. pestis* into the dermis while attempting to feed on a mammalian host. The bacteria are able to rapidly disseminate from the flea bite site to the draining lymph node to cause bubonic plague. After extensive multiplication in the lymph node, the bacteria spread systemically. The high bacteremia level required to infect fleas is typically fatal to the vertebrate host [1], [2].

Adaptation to bloodfeeding on vertebrate hosts has independently evolved many times in the arthropods [3], [4], and in each case the arthropod had to overcome the hemostatic and other defense efforts of its host. This is accomplished primarily by a multitude of pharmacologically active molecules present in the saliva that are injected into the bite site. Arthropod saliva contains a diversity of anti-hemostatic, anti-inflammatory, and immunomodulatory effectors [5]–[8]. Vector-borne pathogens are introduced into a unique microenvironment in the skin that includes this salivary cocktail. It is now well-established that the natural transmission route can influence infection dynamics and differs from needle-injection models. For example, injection of *Leishmania* spp. with sand fly salivary gland extract into naïve mice leads to increased infectivity, higher parasite burdens and increased pathology compared to needle inoculation of parasites alone [9]–[11]. Vector feeding or vector salivary gland extract is known to enhance infectivity of other arthropod-borne diseases, including bacteria [12], viruses [13]–[15], and parasites [16].

Furthermore, exposure to vector saliva in uninfected bites results in an immune response to salivary components, and this can affect transmission and pathogenesis when the animal is later fed upon by an infected vector. In some cases, a history of exposure to uninfected bites can lead to protection. For example, mice previously exposed to uninfected sand fly bites are more resistant to cutaneous leishmaniasis [17]. Immunity to salivary components from past exposures was also shown to inhibit transmission of *Borrelia burgdorferi* by *Ixodes scapularis* [18] and transmission of *Plasmodium yoelii* by *Anopheles stephensi* [19]. With mosquito transmission of West Nile virus (WNV) and tsetse transmission of *Trypanosoma brucei*, prior exposure to vector saliva exacerbated disease [20], [21]. However, in other studies of mosquito transmission of WNV or *Plasmodium*, and tick transmission of Lyme disease spirochetes, prior vector exposure had no obvious effect [22]–[24].

The salivary proteins of the rat flea *Xenopsylla cheopis* and the cat flea *Ctenocephalides felis* have been characterized [25], [26]. In addition to known anti-hemostatic and anti-inflammatory effectors such as apyrase and adenosine deaminase enzymes, esterase, Antigen-5 family proteins, and antimicrobial peptides commonly found in the saliva of other blood-feeding arthropods, flea saliva contains some unique proteins. Most prominently, a large family of closely related acid phosphatases, probably enzymatically inactive, comprises the major protein component of flea saliva; the FS family and several other peptides are also unique to flea saliva [25], [26]. The function of these flea-specific protein families is unknown.

Maintenance of *Y. pestis* depends primarily on flea-rodent transmission cycles. Most rodents harbor a permanent ectoparasitic flea fauna that feed on them daily. However, the immune response to flea saliva and how it might affect plague transmission dynamics has not been characterized. To date, laboratory studies of flea-borne transmission of *Y. pestis* have utilized naïve rodents with no previous exposure to fleas, which is not the natural situation. In this study, we examined the effect of flea saliva on early events in pathogenesis in an intradermal injection model of bubonic plague and characterized the murine immune response to flea saliva. We also evaluated whether pre-exposure to uninfected flea bites and pre-existing immunity to components of flea saliva influence the transmission and disease progression of flea-borne plague.

## Methods

### Bacteria

The fully virulent wild-type *Y. pestis* strain 195/P [27] was used in all experiments. Bacteria were grown in brain-heart infusion broth overnight at 28°C, transferred into LB broth and grown for 24 h at 28°C without aeration. The culture was brought to 20% glycerol and stored in aliquots at −80°C. The titer of aliquots used for injections was tested periodically by limiting dilution on tryptose blood agar plates in triplicate and there was no change in colony forming units (CFU)/ml of the stock over the course of the study.

### Ethics statement

Specific-pathogen-free, 6–12 week old female C57BL/6 mice (Harlan Laboratories) were used for all experiments. All experiments involving animals were approved by the Rocky Mountain Laboratories, National Institute of Allergy and Infectious Diseases, National Institutes of Health Animal Care and Use Committee and were conducted in accordance with all National Institutes of Health guidelines (Animal Protocol Approval #2010-48).

### Salivary gland extract (SGE)

Salivary glands were dissected from adult *X. cheopis* fleas in sterile cell culture grade PBS and transferred to tubes containing PBS, two pairs of salivary glands/µl, and stored at −80°C. Salivary glands were subjected to four freeze/thaw cycles and sonicated on ice (50% power, 5 s on/off pulses for 2 min using a Vibracell VCX130, Sonics and Materials, Newtown, CT). The SGE was cleared by centrifugation (8000× *g* for 5 min) and total protein quantitated by Qubit Quant-iT Protein Assay Kit (Invitrogen, Carlsbad, CA). Halt Protease Inhibitor Cocktail (Thermo Fisher Scientific, Atlanta, GA) was added according to manufacturer's recommendation and SGE was stored at −80°C.

### Flea feeding on mice

*Xenopsylla cheopis* fleas were from laboratory colonies and maintained as previously described [28]. Capsules used to contain fleas while feeding on mice were constructed by cutting the needle end from 10 ml plastic syringes and covering the open end with nylon mesh. The plunger was removed to put fleas into the syringe and replaced to contain them. Twenty-five fleas starved for 4–7 days were placed in a feeding capsule. Mice were anesthetized by subcutaneous injection of ∼70 µg/3 µg ketamine/xylazine per gram body mass. The mesh side of a feeding capsule was secured with tape on the ear pinna of anesthetized mice and fleas were allowed to feed for 30–40 min. Alternatively, a small patch of fur was shaved from the side of the mouse's abdomen and the flea capsule secured with tape over the skin for feeding.

### Naïve mouse challenges

Aliquots of *Y. pestis* were thawed and diluted in PBS to the desired concentration. There were four treatment groups: 1) mice injected intradermally (id) in the ear pinna with ∼250 CFU *Y. pestis* in a total volume of 10 µl (*Y. pestis*-only group); 2) mice that received just the flea feeding procedure described above (fleas-only group); 3) mice that received flea feeding on the ear followed immediately by id injection in the same ear with ∼250 CFU *Y. pestis* in a total volume of 10 µl (fleas + *Y. pestis* group); and 4) mice injected id in the ear with 10 µl PBS (control group). Samples of 5–10 mice from each group were euthanized at 3, 6, 12, and 24 h post-infection (pi). Ears were collected into tubes with 70% EtOH [29]. Superficial parotid lymph nodes (using the nomenclature of Van den Broeck et al [30]) were collected into tubes with 2 ml PBS without Ca^++^ or Mg^++^.

In a separate experiment, fleas were allowed to feed both on the ear and side of naïve mice as described above. At 3, 6, 12, 24 and 48 h after feeding, three mice were euthanized. The ear and a skin biopsy from the feeding site on the side of each mouse was collected and fixed in 10% neutral buffered formalin (NBF) for histological staining. The contralateral ear and skin biopsy were taken as controls.

### Isolation of cells

Ears were removed from EtOH and blotted dry. Ears were carefully peeled apart, separating the two skin layers, and floated dermal side down in a 6-well non-tissue culture treated plate. Wells contained 3 ml RPMI medium (Sigma, Atlanta, GA) with 25 mM HEPES pH 7.5, 1.5% NaHCO_3_, 50 µg/ml DNAse I (Worthington Biochemical Corporation, Newark, NJ) and 26 U/ml Liberase TM (Roche Diagnostics, Chicago, IL). Preparation of single cell suspensions from ear and superficial parotid lymph node samples and determination of bacterial load numbers were done as previously described [31].

### Flow cytometry

Aliquots of 50 µl of single cell suspensions from each sample were dispensed into 96-well round bottom microtiter plates and stained with 1∶200 dilutions of antibodies (BD Pharmingen or eBioscience): anti-Ly-6G (clone 1A8, FITC labeled), anti-CD11b (clone M1/70, labeled with phycoerythrin-Cy7), and anti-F4/80 (clone BM8, allophycocyanin labeled). Rat IgG2a and IgG2b were used as isotype controls. Cells were stained for 30 min at 4°C, spun at 650× g for 1 min and fixed with IC Fixation Buffer (eBioscience, San Diego, CA) for 1 h at 4°C. Cells were spun at 650× g for 1 min and resuspended in PBS with 1% fetal bovine serum. Cell phenotype data were acquired on a Partec CyFlow ML flow cytometer and analyzed with FloMax (Partec) and FloJo (Tree Star) software. Gating strategies were as previously described [31]. Neutrophils were defined as Ly-6G^+^F4/80^−^. Neutrophils that expressed high levels of CD11b [29] (CD11b^high^) were defined as activated neutrophils [31]–[35]. Macrophages were defined as F4/80^+^Ly6G^−^ cells.

### Sensitization of mice to flea bites

Three groups of five mice each received three different flea exposure regimens for ten weeks. Group A mice were fed on by 25 fleas once per week; Group B were exposed to 50 fleas once per week; and Group C 25 fleas twice per week. All flea feeds were done on a shaved area on the side of the mouse. After each exposure, fleas were individually examined under a dissecting microscope, and the number of fleas that had fed (containing fresh blood in the midgut) was recorded. Mice were tracked individually to determine the total number of flea bites for each mouse. After five weeks of exposure, blood samples were taken to collect serum for detection of antibody to salivary proteins (5-week sera). At the end of the 10-week exposure period, mice were exposed a final time on each ear, 25 fleas per ear; 12 h later the mice were euthanized. For each mouse, one ear was removed and processed for flow cytometry analysis as described; the other ear and a skin biopsy from the flea feeding site on the side of the mouse was fixed in NBF for histological staining (Group A mice were not sampled for histology). Blood samples were taken to collect serum for detection of antibody to salivary proteins (final sera).

### Histology

Tissue samples fixed in NBF were embedded in paraffin, sectioned, and stained with hematoxylin and eosin. For each tissue sample, 4–12 sections were examined and subjectively categorized by a board-certified veterinary pathologist (D. Gardner) and assigned a numerical inflammation severity score from 0 to 2: 0 = within normal limits (i.e., not different from unbitten controls); 1 = minimal inflammation: very few to low numbers of inflammatory cells in the dermis and/or subcutis; 2 = mild inflammation: low to moderate numbers of inflammatory cells within the dermis and/or subcutis; inflammatory cells are detectable at 4–10X magnification and may aggregate together.

### Detection of antibody to flea salivary proteins

Sera collected from flea-exposed mice were screened for IgG response to flea salivary proteins by Western blot. SGE (5 µg/lane) was separated by SDS-PAGE on 4–20% polyacrylamide gradient gels and transferred to 0.2 µm nitrocellulose using a Criterion blotting apparatus (BioRad, Richmond, CA). Blots were blocked in 5% dried skim milk in Tris-buffered saline (TBS) overnight at 4°C. Blots were cut into strips and incubated with serum samples diluted 1∶250 in 2% dried skim milk in TBS with 0.05% Tween 20 for 2 h at room temperature with gentle agitation. Blots were washed in TBS-Tween then incubated with goat anti-mouse IgG (Invitrogen) at 1∶10,000 for 1 h at room temperature. Blots were washed again in TBS-Tween and developed using the BCIP/NBT liquid substrate (Sigma Life Science, Atlanta, GA). A polyclonal antibody raised to SGE in mice (prepared by Lampire Biological Laboratories, Pipersville, PA) was used at 1∶10,000 as a positive control; naive mouse serum at 1∶250 served as a negative control.

To quantitate the IgG response to SGE, an ELISA was developed. Costar 96-well flat bottom high-binding EIA plates (Fisher Scientific, Pittsburg, PA) were coated with SGE in 0.05 M carbonate/bicarbonate buffer, pH9.6 (100 µl/well at 0.5 ng/µl) overnight at 4°C. Plates were blocked with 5% dried skim milk in PBS-0.05% Tween-20 for at least 2 h at 28°C, then incubated with unknown sample sera or naïve mouse serum at 1∶250 in 2% dried milk in PBS-Tween for 2 h at 28°C. After washing with PBS, goat anti-mouse IgG horseradish peroxidase conjugate (Thermo Scientific) was added at 1∶20,000 in 2% dried milk in PBS-Tween and incubated for 1 h at 28°C. Plates were washed with PBS-Tween and developed using the Ultra TMB-ELISA substrate (Thermo Scientific). Color development was stopped with 2 M H_2_SO_4_ and absorbance of wells read at 450 nm on a Synergy 2 microplate reader (Bio Tek Instruments, Winooski, VT). Sera were tested in triplicate. For each ELISA run a standard curve was built using serial 2-fold dilutions (1∶1250–1∶1280K) of the polyclonal anti-SGE serum. The 1∶2500 dilution was arbitrarily assigned a value of 10,000 antibody units (U). A standard curve of log_10_(U) plotted against A_450_ was fitted to the 4-parameter logistic regression model in GraphPad Prism v.5.01 (GraphPad Software, Inc., La Jolla, CA), with the hill slope constrained to 1.0 and bottom parameter constrained to the average negative control value. The log_10_(U) of unknown sera was interpolated from the standard curve. Sera with values > mean of the negative controls +2 SD were considered positive. Similar ELISAs were developed to quantitate the IgG1, IgG2a, IgG2c, IgM, and IgE responses. Anti-mouse secondary antibodies to these antibody isotypes were obtained from Thermo Scientific (α-IgM, α-IgE, and α-IgG2c) or Jackson ImmunoResearch (α-IgG1 and α-IgG2a).

### IgG response in wild rats to *X. cheopis* salivary proteins

Sera from 20 wild *Rattus norvegicus* from Los Angeles, CA were obtained during surveys conducted in 2003. Rats were combed for ectoparasites and the only species found was *X. cheopis*. Sera were tested for antibodies against SGE by Western blot as described above.

### Challenge experiments using infective (blocked) fleas

Two groups of 20 mice each received contrasting exposure regimens to uninfected fleas. One group was fed on by 25 fleas once per week for 5 weeks (low exposure); the second group was fed on by 25 fleas twice per week for ten weeks (high exposure). After five weeks of exposure a serum sample was taken from both groups. *X. cheopis* fleas were infected with *Y. pestis* by using an artificial feeding device [36] and monitored for proventricular blockage as previously described [37]. Three blocked fleas were used to challenge individual mice for 1 h on a shaved area on the side of the abdomen. After the 1 h feeding period the fleas were examined microscopically and the number that attempted to feed (fresh blood in the esophagus) was recorded. Naïve (not pre-exposed to fleas) mouse controls were similarly challenged by blocked fleas. Mice were monitored for the appearance of illness (lethargy, ruffled fur, hunched posture, reluctance to respond to external stimuli) and euthanized; time to terminal disease in hours was recorded. Triturated spleen and blood samples recovered from each mouse after euthanasia were cultured on blood agar plates to confirm *Y. pestis* infection. Final sera were taken from all survivors at the end of the experiment.

### Statistical analyses

Bacterial loads in infected mice and IgG responses in mice from the challenge experiments were compared using Student's t test. The association between bacterial loads and neutrophil recruitment in the ear and draining lymph node, and the association between number of flea bites and IgG response, was tested by Pearson correlation analysis. For flow cytometric data, groups were compared by Kruskal-Wallis nonparametric ANOVA, followed by Dunn's multiple comparison test to detect differences between treatments or timepoints. Survival curves in the challenge experiment were compared by the Mantel-Cox logrank test. Analyses were done using GraphPad Prism software (version 5.01).

## Results

### *X. cheopis* flea bites induce mild inflammation in the skin of naive mice

**Figure 1:**
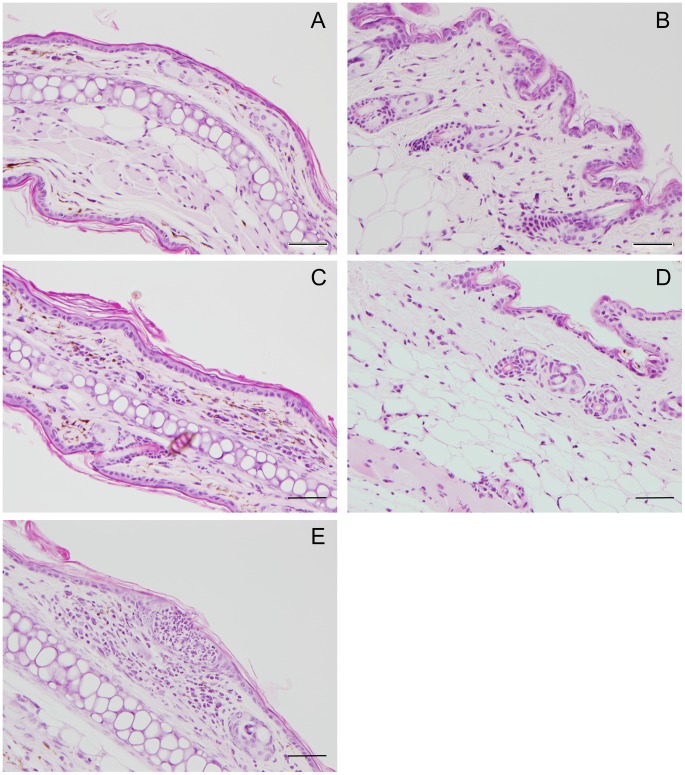
Minor histological changes in skin of naive mice within 48 h of exposure to flea bites. Representative skin sections from ear (**A**, **C**) and abdomen (**B**, **D**) collected from mice 24–48 h after flea bites (**C, D**) or from normal unbitten control mice (**A, B**). Flea feeding induced minimal inflammation (score = 1) compared to control ear (score = 0). Panel E shows a focus of neutrophil infiltration found in one mouse 24 h after flea feeding (score = 2). Ten of 15 mice were indistinguishable from the unfed controls. See Methods for scoring criteria. Scale bars = 50 µm.

Insect bites often cause local cutaneous inflammatory reactions, ranging from mild erythema to papule formation and edema, largely determined by salivary components [38]–[41]. The only obvious dermal sign after 22–28 *X. cheopis* fleas fed on the ear and abdominal skin of naïve mice were occasional small discrete erythematous spots, with no swelling or papule formation. Figure 1 shows representative histological examples of inflammation observed in mouse skin within 48 hrs of flea feeding. Three mice showed minimal inflammation (severity score = 1) in the dermis of the ear (Fig. 1C) or abdomen (Fig. 1D) compared to controls (Fig. 1A and B). One mouse had a focus of moderate inflammation (severity score = 2) in the ear (Fig. 1E). In ten of 15 mice examined, skin from flea-fed areas was indistinguishable from unbitten control skin.

### Exposure of naïve mice to flea bites does not affect the kinetics of innate immune cell recruitment to *Y. pestis* infection or bacterial survival

Arthropod saliva can modulate the migration and defense responses of innate immune cells. Consequently, in naïve animals (with no prior exposure to the vector), injection of a pathogen into the skin where an uninfected vector has recently fed, or coinjection of the pathogen with vector SGE, often results in enhanced disease progression compared to injection of the pathogen alone [7], [9], [12]–[16]. We compared neutrophil and macrophage recruitment following id injection of fully virulent, wild-type *Y. pestis* 195/P into the ear of two groups of naïve mice, one of which had received 11–22 flea bites on the ear immediately before injection (fleas + *Y. pestis* group) and one which had not (*Y. pestis*-only group). Two other groups of mice received flea bites only or id injection of PBS.

**Figure 2:**
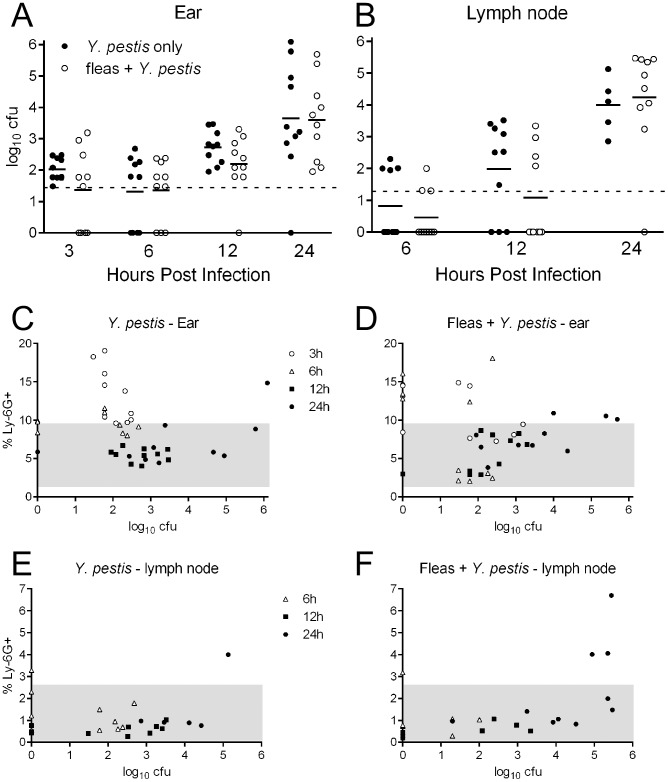
Injection of *Y. pestis* at a flea feeding site does not affect bacterial survival or dissemination kinetics in naïve mice. Mice were infected intradermally in the ear with ∼250 CFU *Y. pestis*, with or without prior flea feeding at the injection site. Bacterial load in the ear (**A**) and draining superficial parotid lymph node (**B**) were not significantly different at any timepoint after infection. Horizontal black lines indicate mean CFU per group; dashed lines indicate the lower limit of detection of the assay. A general lack of correlation between bacterial load and neutrophils recruited in the ear (**C, D**) and lymph node (**E, F**) was seen for both the *Y. pestis*-only (**C, E**) and fleas + *Y. pestis* (**D, F**) mouse groups. The shaded areas represent the range of % neutrophils obtained from the PBS injected controls. Each symbol represents an individual mouse. Data are pooled from two independent experiments per timepoint.

The mean ± standard deviation of inocula for all mice infected with *Y. pestis* was 281±62 CFU. Bacterial loads were measured in the ear (Fig. 2A) and draining lymph node (Fig. 2B) of mice at different times after id infection with *Y. pestis*, with and without the presence of flea feeding. The *Y. pestis*-only and fleas + *Y. pestis* groups did not differ significantly from each other in the number of CFU recovered at any timepoint.

**Figure 3:**
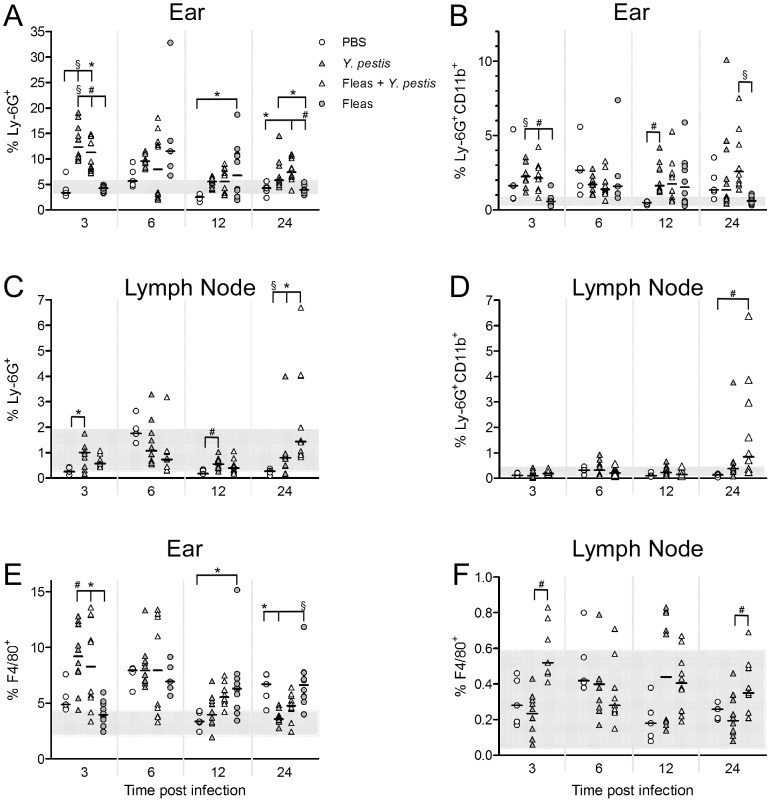
Timecourse of neutrophil and macrophage responses in the ear and draining lymph node of naïve mice. Total neutrophils, expressed as the percentage of Ly-6G^+^ cells out of all events counted (**A**, **C**); activated neutrophils, expressed as the percentage of Ly-6G^+^CD11b^high^ cells out of all events counted (**B**, **D**); and total macrophages, expressed as the percentage of F4/80^+^ cells out of all events counted (**E**, **F**) in the ear and lymph node at different times after infection were determined by flow cytometry. The four different treatment groups are indicated; each symbol represents an individual. Horizontal bars indicate the median and the shaded area represents the range of values measured from a group of 6 uninjected control C57BL/6 mice. Median percentages were compared by Kruskal-Wallis nonparametric ANOVA followed by Dunn's multiple comparison test. *, 0.01<P<0.05; #, 0.001<P = 0.01; §, P = 0.001.

At 3 h pi, both groups of infected mice had significantly greater neutrophil recruitment (total Ly-6G^+^ cells) in the ear than control mice receiving only flea feeding (fleas-only) or an id injection of PBS (Fig. 3A and B). At 6 h pi the number of neutrophils in mice infected with *Y. pestis* (with or without prior flea feeding) decreased, but increased in the fleas-only treatment group. At 12 h pi, the fleas-only group had significantly higher % total neutrophils (P<0.05) compared to the PBS controls (Fig. 3A). By 24 h pi, neutrophils returned to PBS control level in the fleas-only group, but showed a variable response in the *Y. pestis* infected groups, with some mice similar to controls and some mice showing an influx of activated (Ly-6G^+^CD11b^high^) neutrophils. Overall, the *Y. pestis*-only and fleas + *Y. pestis* groups had the same kinetics: both showed early neutrophil recruitment at 3 h, which decreased significantly through 12 h pi and began to increase at 24 h as disease progressed. These treatments did not differ significantly from each other at any timepoint. In contrast, the fleas-only treatment showed a significant increase in neutrophils peaking at 6–12 h and declining to control levels by 24 h.

In the draining lymph node, there were some differences among treatments in total neutrophils, but these mostly fell within the range of values seen in completely unmanipulated controls (Fig. 3C and D). The exception to this was at 24 h, where some individuals in the *Y. pestis*-only and fleas + *Y. pestis* groups showed an increased influx of neutrophils, while other individuals were still in the range of control mice. This variation likely reflects differences in the progression of bubonic plague among individual mice [29]. In the fleas-only group, neutrophils in the lymph node remained in the range of control mice throughout the experiment.

The reduction in neutrophils before 24 h pi in mice infected with *Y. pestis* is represented again in the graph of % total neutrophils vs. bacterial load (Fig. 2). In the ear, at 3 and 6 h, there was detectable neutrophil recruitment which was reduced to PBS control level by 12 h. This occurred whether or not mice were exposed to flea bites before infection (Fig. 2C and D). Only after bacterial loads exceeded 4 log_10_ in ear or lymph node did we again observe some mice with neutrophil responses greater than PBS controls. There was no correlation between log_10_ CFU and % Ly-6G^+^ cells in the ear in either treatment (*Y. pestis*-only: r = −0.21, P = 0.21; fleas + *Y. pestis*: r = −0.17, P = 0.28). In the draining lymph node, neutrophil numbers did not begin to exceed those in PBS controls until 24 h pi, even in mice with 4–5 log_10_ bacteria (Fig. 2E and F), with or without flea feeding. In the *Y. pesti*s-only group, there was no correlation between log_10_ CFU and % Ly-6G^+^ cells (r = 0.08, P = 0.70). In the fleas + *Y. pestis* group there was a significant positive correlation between log_10_ CFU and % Ly-6G^+^ cells (P = 0.007), but with a low r^2^ (0.354). The correlation was dependent on the three mice at 24 h pi with an influx of neutrophils well above those seen in PBS controls (Fig. 3D); removing these outliers resulted in a non-significant correlation (P = 0.061).

In both groups infected with *Y. pestis* macrophage recruitment in the ear was similar to that of neutrophils: at 3 h pi these mice had significantly greater macrophage recruitment compared to fleas-only mice (P = 0.002), which decreased to PBS control level by 12 h pi (Fig. 3E). The *Y. pestis*-only and fleas + *Y. pestis* groups did not differ significantly from each other at any timepoint. In the fleas-only group, the presence of macrophages was not different from controls at 3 h pi but rose significantly at 6 h pi (P<0.01) and remained elevated for the rest of the experiment, significantly greater than the *Y. pestis*-only group at 24 h pi (P<0.001). In the lymph node, there were some statistically significant differences between groups, but macrophages made up a very small percentage of cells, generally less than 1% in all treatments (Fig. 3F). Also, median percentages of F4/80^+^ cells of all treatments were within the range of values seen in unmanipulated mouse controls.

### Immune response of mice and rats to *X. cheopis* flea saliva following prolonged exposure to flea bites

**Figure 4:**
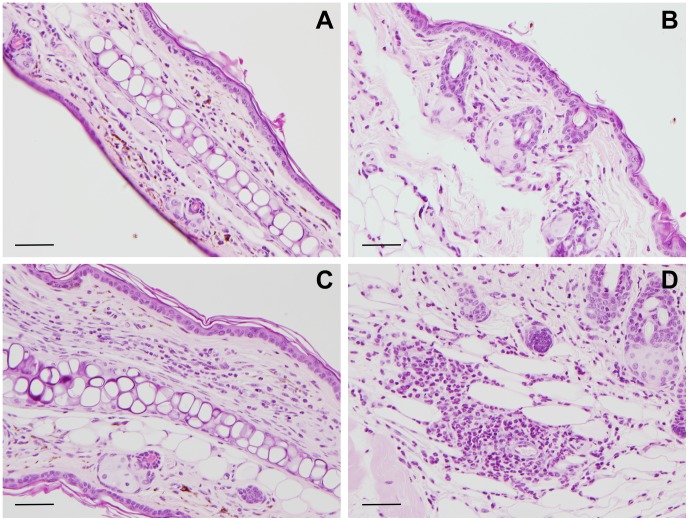
Histological response to flea bites in skin of mice previously exposed to fleas for ten weeks. Representative examples of skin sections from ear (**A**) and abdomen (**B**) showing minimal inflammation (score = 1), and from ear (**C**) and abdomen (**D**) showing mild inflammation (score = 2). Samples were from mice that had been exposed to flea bites for 10 weeks (Group B and C mice, see Table 1 and Methods for details). Four of 10 and 2 of 10 ear samples had a score of 1 or 2, respectively; 5 of 10 and 2 of 10 abdominal skin samples had a score of 1 or 2, respectively. All other samples were indistinguishable from controls. See Methods for scoring criteria. Scale bars = 50 µm.

A second goal of this study was to determine if an anamnestic or hypersensitivity response develops to flea saliva and if it affects flea-borne plague transmission dynamics. Three groups of mice were fed upon by uninfected *X. cheopis* flea bites throughout a 10-week period according to the regimen shown in Table 1. Histological examination of Groups B and C revealed no evidence of a hypersensitivity reaction as described in other arthropod-host systems [42], [43]. After 10 weeks, four out of five Group C mice showed minimal inflammation (severity score = 1) in the ear (Fig. 4A) and abdominal skin (Fig. 4B), or were indistinguishable from control tissue (severity score = 0). One mouse in this group showed mild inflammation (severity score = 2) in the skin 12 h after flea feeding (Fig. 4D). Group B mice had a similar pattern: two mice had a score of 2 in the ear (Fig. 4C) and two mice had a score of 2 in the skin. In addition, one out of five mice had a score of 1 in the ear or skin. Throughout the course of flea exposure, mice were observed at 1 and 2 days after feeding, and none showed any swelling or delayed type hypersensitivity response at the feeding site. Some had transient reddening of the skin which did not last from one flea feeding episode to the next. Overall, mice with a history of flea exposure showed little inflammation or hypersensitivity in response to flea feeding.

**Table 1:** Antibody responses of mice to flea salivary gland extract after exposure to uninfected flea bites.

			IgG response^a^				IgM response^a^	
Mice used for	Exposure history^b^	Total flea bites^c^ (mean ± s.d.)	5-week sera		10-week sera		10-week sera	
			No. +	log_10_ (U)	No. +	log_10_ (U)	No. +	log_10_ (U)
Sensitization								
Group A	25 fleas once/wk for 10 weeks	289±14	0/5	0	3/5	2.97±0.9	0/5	0
Group B	50 fleas once/wk for 10 weeks	416±46	1/5	1.46	4/5	2.82±0.3	2/5	0.69±0.03
Group C	25 fleas twice/wk for 10 weeks	441±34	4/5	2.11±0.6	5/5	3.03±0.4	1/5	0.62
Challenge								
Low exposure	25 fleas once/wk for 5 weeks	110±10	0/20	0	7/14	2.11±0.6	1/14	1.55
High exposure	25 fleas twice/wk for 10 weeks	523±20	19/20	2.47±0.8	16/17	2.33±0.7	0/17	0

a ELISA results; number positive out of total (No. +) and the mean units of antibody (log_10_ (U) ± s.d.) of the positive samples are shown. Positive samples defined as having a mean log_10_ (U)>2 s.d. above negative control sera.

b Fleas fed on the abdomen of the mice. At the end of their 10-week exposure period, Groups A, B and C mice used to evaluate sensitization also received a final exposure of 25 fleas on the ear 12 hours prior to euthanasia.

c mean total cumulative bites received per mouse.

All 5-week samples were negative for IgM and all samples were negative for IgE.

**Figure 5:**
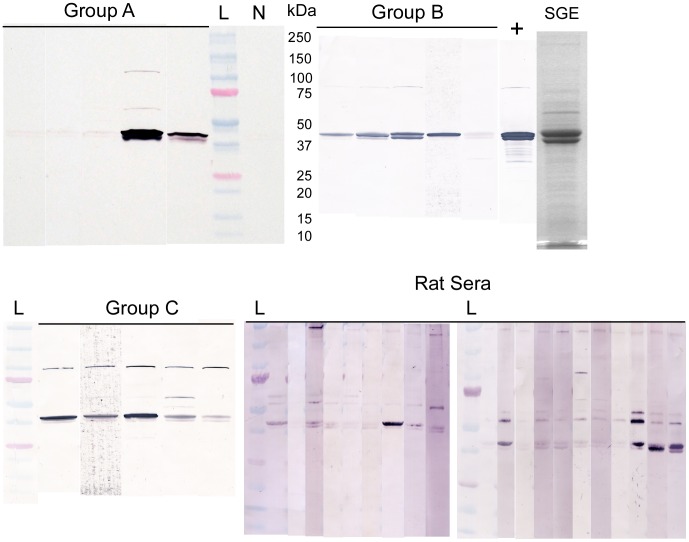
Serum IgG responses to flea SGE in mice and rats previously exposed to flea bites. (**A**–**C**) Immunoblots of *X. cheopis* SGE probed with sera from 5 mice previously exposed over a ten-week period to: (**A**) 25 fleas once per week, (**B**) 50 fleas once per week, or (**C**) 25 fleas twice per week. (Mouse groups A, B, and C, respectively; see Table 1 for details.) (**D**) Immunoblot results from sera of 20 wild *Rattus norvegicus* trapped in Los Angeles, where rats are naturally infested with *X. cheopis* fleas. Brackets indicate the position of the 36–45 kDa phosphatase-like proteins, the major component of flea saliva. All sera were tested at a dilution of 1∶250, negative control serum from an unexposed C57BL/6 mouse at 1∶250; + = positive control mouse serum at 1∶10,000; SGE = Coomassie stained SDS-PAGE of salivary gland extract. Molecular weights (kDa) of protein standards (M) are indicated at left.

Mice with the lowest exposure to flea bites (Group A) had inconsistent seroconversion, with three out of five mice showing little or no IgG response to *X. cheopis* SGE in immunoblots (Fig. 5). The higher exposure regimens (Groups B and C) generated more consistent antibody responses to SGE (Fig. 5, Table 1). There was an increase both in numbers of positive mice and in the magnitude of the IgG response between sera collected after 5 weeks of exposure to fleas and final sera taken after ten weeks of exposure. There was a significant correlation (P = 0.001, r^2^ = 0.22) between the total number of flea bites received by an individual mouse and its IgG log_10_(U). Most of the IgG response was directed to the prominent protein band of 40–43 kDa, which corresponds to the phosphatase family proteins [25], the major component of *X. cheopis* SGE. (Fig. 5). Reactivity to a ∼100 kDa SGE antigen was also seen in mice with the highest exposure to flea bites (Group C). Results of IgG1, IgG2a and IgG2c-specific ELISA showed antibody against SGE was highly skewed toward the IgG1 subtype with very little production of IgG2a or IgG2c (Table 2), indicating flea feeding stimulated a Th2-biased response in mice. Minimal IgM responses were found in a few of the final sera from Groups B and C (Table 1), and none of the sera tested had detectable IgE by ELISA.

**Table 2:** Th2 bias of antibody response to flea saliva in mice exposed to flea bites.

	IgG1 response^a^				IgG2a response^a^				IgG2c response^a^			
	5-week sera		10-week sera		5-week sera		10-week sera		5-week sera		10-week sera	
Mice used for^b^	No. +	log_10_ (U)	No. +	log_10_ (U)	No. +	log_10_ (U)	No. +	log_10_ (U)	No. +	log_10_ (U)	No. +	log_10_ (U)
Sensitization												
Group A	0/5	0	3/5	3.58±1.7	1/5	0.87	0/5	0	0/5	0	0/5	0
Group B	1/5	1.23	4/5	3.27±0.3	0/5	0	0/5	0	0/5	0	1/5	1.21
Group C	1/2	0.84	5/5	2.99±0.6	0/2	0	0/5	0	0/5	0	1/5	0.68
Challenge												
Low exposure	0/20	0	7/14	1.98±0.7	0/18	0	1/14	0.34	0/18	0	1/14	0.94
High exposure	19/19	2.51±0.9	17/17	3.12±0.9^c^	0/19	0	9/17	0.62±0.4	6/19	1.52±0.9	6/17	1.78±0.6

a ELISA results; number positive out of total (No. +) and the mean units of antibody (log_10_ (U) ± s.d.) of the positive samples are shown. Positive samples defined as having a mean log_10_ (U)>2 s.d. above negative control sera.

b See Table 1 for exposure history of the mouse groups.

c Three sera in this group were above the range of the standard curve.

The IgG response to flea saliva was also surveyed in the sera of wild brown Norway rats (*R. norvegicus*) trapped in Los Angeles, where the sole ectoparasitic flea species is *X. cheopis*. As with the C57BL/6 mice, rat serum IgG reactivity to SGE proteins was variable, with the flea phosphatases being immunodominant (Fig. 5). A band at about 55 kDa seen in three mice also appeared frequently in the rat samples; the 100 kDa band was clearly present in only one of the rats. Rats were trapped during August and November, representing seasons of high and low flea index, respectively. There was no discernable correlation between immunoblot results and date trapped or rat age (adult, subadult, or juvenile) or weight (55–370 g).

### Long-term prior exposure to fleas attenuates the dermal inflammatory response to flea bites

**Figure 6:**
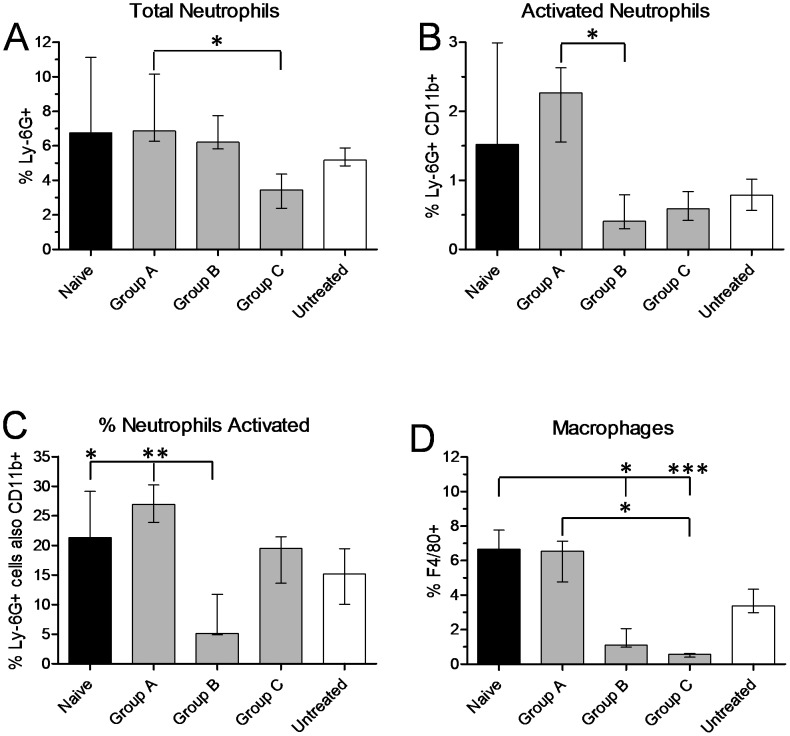
Long-term exposure to fleas attenuates the inflammatory response to flea bites. Flow cytometry quantitation of total neutrophils, expressed as the percentage of Ly-6G^+^ cells of all cells counted (**A**); activated neutrophils, expressed as the percentage of Ly-6G^+^CD11b^high^ cells of all cells counted (**B**); % neutrophils activated, expressed as the percentage of all Ly-6G^+^ cells also expressing high levels of CD11b (**C**); and macrophages, expressed as the percentage of F4/80^+^ cells of all cells counted (**D**). Graphs compare a naïve group of 10 mice exposed to fleas for the first time to mice that had received three different flea exposure regimes (Groups A, B, C; see Methods and Table 1; n = 5 per group). The untreated group represents 6 mice that had never been exposed to fleas. Bars show median percentages with interquartile range. Median percentages were compared by Kruskal-Wallis nonparametric ANOVA followed by Dunn's multiple comparison test. *0.01<P≤0.05, **0.001<P≤0.01, ***P<0.001.

In naive mice after a first exposure to fleas, recruitment of neutrophils and macrophages peaked at 6–12 hours in ear tissue (Fig. 3). At the end of their 10-week flea exposure regimen (Table 1), Group A, B and C mice were exposed to flea bites a final time on the ear. Twelve hours later the presence of neutrophils and macrophages was assessed by flow cytometry and compared to the naïve mouse 12 h timepoint (Fig. 3). Total neutrophils (Ly-6G^+^ cells) from Group C mice, a high exposure treatment, was significantly less (P<0.05) than the response seen in the low exposure Group A (Fig. 6A). In addition, fewer activated neutrophils (Ly-6G^+^CD11b^high^ cells) were present in Groups B and C than in Group A, with a statistically significant difference between Group A and B (P<0.05, Fig. 6B). Figure 6C shows % neutrophils activated, the percentage of Ly-6G^+^ cells also CD11b^high^. Group B was significantly less than both Group A (P<0.01) and the naïve mice group (P<0.05). Finally, mice with higher exposure to fleas (Groups B and C) had a much lower macrophage response than Group A (vs. Group C: P<0.05) or naïve mice (vs. Group B: P<0.05; vs. Group C: P<0.001) (Fig. 6D). Overall this indicates a reduction in neutrophil and macrophage recruitment 12 h after flea feeding in experimentally exposed mice compared to naive mice receiving flea bites for the first time.

### Prior repeated exposure of mice to flea bites does not affect transmission rate of *Y. pestis* by flea bite or disease progression

**Figure 7:**
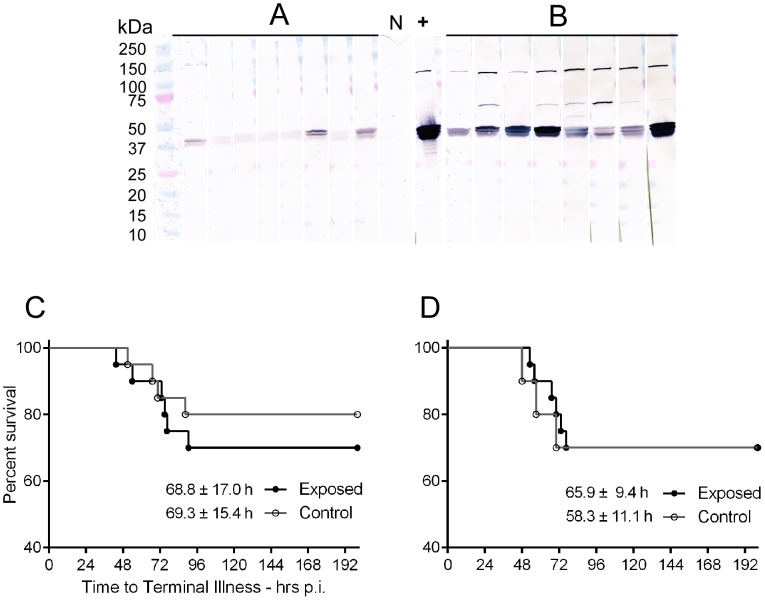
Previous exposure to uninfected flea bites does not affect the incidence or progression of bubonic plague in mice challenged by infective (blocked) fleas. Immunoblots showing IgG responses to *X. cheopis* salivary gland extract of 8 representative individuals from each of the low-exposure (**A**, 25 fleas once per week for 5 weeks) and high-exposure (**B**, 25 fleas twice per week for 10 weeks) group (see Table 1). Molecular weights (kDa) of protein standards are indicated at left; bracket at right indicates the position of the 36–45 kDa phosphatase-like SGE proteins. Sera were used at a dilution of 1∶250, negative control serum from an unexposed C57BL/6 mouse at 1∶250; +, positive control mouse serum at 1∶10K. The incidence and time to development of terminal plague in mice challenged with *Y. pestis*-blocked fleas after low- (**C**) or high-level (**D**) prior exposure to uninfected fleas were not significantly different from naive mouse controls. Inset numbers indicate time to terminal disease in hours (mean ± SD).

To determine if a history of exposure to flea bites and the resulting immune response to flea saliva affects transmission dynamics, progression, or severity of disease, we challenged naïve and sensitized mice with *Y. pestis* by the natural, flea-borne infection route. Two such experiments were done (Table 3), comparing naïve mice to mice exposed to 25 fleas once per week for 5 weeks (low exposure group) or twice per week for 10 weeks (high exposure group). By immunoblot, mice from the low and high exposure groups had qualitatively very different serum IgG levels to *X. cheopis* SGE (Fig. 7A, B). These sera were also quantitatively analyzed by ELISA (Table 1). The low and high groups differed significantly in log_10_(U) values (t-test, P = 0.004), and represent two contrasting treatment levels for challenge with *Y. pestis* by flea bite. The low and high exposure mice were challenged in tandem with naïve control mice by allowing three fleas that had been infected and become blocked with *Y. pestis* 195/P to feed on them. Table 3 shows data from individual mice in the challenge experiments. Survival curves (Fig. 7C, D) of the low and high group did not differ from their naïve control group by logrank test (low: P = 0.50, high: P = 0.92). Exposed mice also did not differ in time to terminal disease compared to control mice (t-test; low: P = 0.96, high: P = 0.32).

**Table 3:** Disease outcome of individual mice challenged by blocked flea bites.

Experiment 1						Experiment 2					
Naïve control group^a^			Low exposure group^a^			Naïve control group^a^			High exposure group^a^		
mouse	blocked flea bites^b^	TTD^c^	mouse	blocked flea bites^b^	TTD^c^	mouse	blocked flea bites^b^	TTD^c^	mouse	blocked flea bites^b^	TTD^c^
1	2	S	1	2	S	1	3	70.0	1	1	S
2	2	70.5	2	1	S	2	1	S	2	3	S
3	3	88.5	3	3	S	3	1	48.0	3	2	73.0
4	2	S	4	2	73.0	4	1	S	4	1	S
5	1	S	5	3	90.5	5	1	S	5	1	70.0
6	1	S	6	2	S	6	1	S	6	2	S
7	1	S	7	1	S	7	1	57.0	7	1	S
8	3	S	8	1	S	8	3	S	8	2	53.0
9	1	S	9	1	S	9	1	S	9	1	S
10	2	S	10	1	S	10	1	S	10	1	S
11	2	67.0	11	1	S	11			11	2	S
12	1	S	12	1	76.5	12			12	1	S
13	2	S	13	1	54.0	13			13	1	S
14	1	S	14	1	S	14			14	1	S
15	1	S	15	2	S	15			15	1	S
16	2	S	16	1	75.0	16			16	3	56.0
17	1	S	17	1	43.5	17			17	1	76.5
18	3	51.0	18	2	S	18			18	2	67.0
19	1	S	19	2	S	19			19	1	S
20	1	S	20	2	S	20			20	1	S
mean	1.65	69.2		1.55	68.8	mean	1.40	58.3		1.45	65.9
median	1.50	68.8		1.00	74.0	median	1.00	57.0		1.00	68.5
SD	0.74	15.4		0.69	17.0	SD	0.84	11.1		0.69	9.4

a naive mice had no prior exposure to flea bites; low- and high-exposure mouse groups had received uninfected flea bites for 5 or 10 weeks, respectively, prior to challenge (see Table 1).

b number of the three blocked fleas used for challenge that attempted to feed.

c time to terminal disease; S = survivor (no disease).

## Discussion

The immune sensitization of humans and other mammals to mosquito, sandfly, and flea bites often follows a characteristic five-stage sequence that evolves with repeated exposure [42], [44]. The initial bites experienced by a naïve animal usually do not produce any observable skin reaction (stage I). After a week or so of continued exposure, a delayed-type hypersensitivity response develops, typified by pruritic papules or vesicles that appear ∼24 hours after the bite (stage II). As exposure continues, an immediate-type hypersensitivity response is seen within 30 minutes of the bite, which subsides but is followed by a delayed-type reaction (stage III). With prolonged frequent exposure to bites, the delayed-type response no longer develops, leaving only the immediate-type response (stage IV). Finally, desensitization or tolerance to the saliva develops, with no further skin reactivity (stage V). Histologically, an influx of mononuclear cells is seen at stage II; in later stages neutrophils, eosinophils and basophils are also prominent [45], [46]. Serum IgG and IgE antibodies specific to vector salivary proteins can be demonstrated [47]–[51]. This reactivity syndrome is indicative of an allergic or hypersensitivity response to insect bites. It has been described in guinea pigs [52] and dogs [46] following exposure to the cat flea *Ctenocephalides felis*, and allergic dermatitis in dogs and cats due to flea-bite hypersensitivity is an important veterinary concern [53].

In this study we found that C57/BL6 mice exposed to *X. cheopis* flea bites did not follow the stereotypical pattern described above. During the ten-week period in which a total of 35 mice received an average of 27–56 flea bites per week, no evidence of an immediate- or delayed-type hypersensitivity response was observed. The only dermal sign, regardless of the duration of exposure, was a transient non-papular, non-edematous erythematous area that was sometimes present on the skin immediately after feeding, probably the result of minor blood leakage due to anticoagulant effects of flea saliva. Flow cytometry data showed that flea bites elicited only a mild inflammatory response, evidenced by a small increase in neutrophil and macrophage recruitment that tended to subside after prolonged exposure (Fig. 3, 5, 6). No obvious mononuclear cell, eosinophil, basophil, or mast cell response was ever detected by histopathology (Fig. 1, 4). Identical results were seen in BALB/c mice exposed to ∼20 fleas per week for 10 weeks (Table S1, Fig. S1). Our results with mice are consistent with a previous study of the response of Sprague-Dawley rats (*R. norvegicus*) to *X. cheopis* flea bites over a four-week period, which also reported no obvious skin reaction and only a slight increase in neutrophils and mononuclear cells noted by histopathology [54].

In addition to monitoring the local immune response at the dermal bite site, we also characterized the murine adaptive immune response to flea saliva after prolonged exposure to flea bites. Mice produced antibodies to salivary proteins after different intensities of exposure to *X. cheopis*, with a general trend of increasing IgG with increasing total number of flea bites (Fig. 5; Tables 1, 2). The IgG subtype production (mostly IgG1 with very little IgG2a or IgG2c) indicates a strong Th2 polarization in response to flea saliva. Most studies of exposure to salivary antigens from ticks [55], sand flies [56], tsetse [48], and mosquitoes [57] also have shown a Th2 bias by isotype antibody production and cytokine profile.

Although we were not able to simulate continuous infestation with fleas, as occurs in nature, serum from wild rats collected in Los Angeles, where *X. cheopis* is the only important flea ectoparasite, showed an antibody profile similar to our flea-exposed mouse sera. Thus, the detected murine response is not an artifact of exposure schedule, the use of laboratory-colonized *X. cheopis* or an inbred mouse strain. For both laboratory mice and wild rats, the immunodominant antigen was the family of 36–45 kDa acid phosphatase-like proteins, the predominant component of flea saliva. Antibodies to SGE proteins of ∼55 and ∼100 kDa were also detected. A similar immunoblot profile was observed in mice exposed to cat flea (*C. felis*) bites [58]. The function of the phosphatase-like family is unknown. There are ten identified transcripts, all with amino acid changes in their catalytic sites that presumably eliminate phosphatase activity [25]. However, they all have a basic pI >8.5 and still may be able to bind negatively charged substrates. One possibility is that they bind polyphosphate released by activated platelets [59]. If flea saliva is able to locally deplete polyphosphate this would inhibit platelet aggregation and blood coagulation [60], [61].

An IgE response to salivary components is commonly associated with the allergic response described above to arthropod bites in lab animals as well as natural hosts [47]–[50], [62]. In contrast, we did not detect serum IgE in any of the mice in our experiments, in keeping with the lack of any obvious allergic reaction at the flea bite sites. Similarly, a study of dogs found that animals allergic to flea bites produced high levels of flea antigen-specific IgE, but a group of dogs exposed to fleas constantly from a young age that showed no reaction to flea bites had IgE levels not significantly different from that of unexposed controls [63]. The authors concluded that chronic exposure to fleas resulted in tolerance in these animals to flea allergens.

Overall the innate, adaptive and hypersensitivity immune responses of mice to *X. cheopis* saliva appear to be quite limited. As suggested by Vaughan et al. (1989) with rats [54], mice may have a sort of adaptive tolerance to *X. cheopis* that is not seen in unnatural hosts such as guinea pigs [45]. In addition, because they live in close association with their hosts and require frequent blood meals, *X. cheopis* may have coevolved to not induce resistance in their usual hosts. We observed no reduction in feeding success of fleas used in any of the mouse sensitization trials, even in groups feeding on mice after twenty previous exposures that had strong antibody responses to SGE. This is consistent with the Vaughan et al.(1989) rat study in which no differences in the number of fleas that fed, blood meal size, or flea longevity was observed between fleas that fed on naïve rats compared to fleas fed on rats that had been sensitized to *X. cheopis* [54]. Thus, the immune response of mice and rats to *X. cheopis* appears to be one of tolerance rather than resistance. This can be contrasted to the strong hypersensitivity responses that develop to the bites of sandflies and hard ticks, which act to deter blood-feeding [64]–[67]. Unlike *X. cheopis*, sandflies and most hard ticks do not live in close contact with or feed repeatedly on an individual host.

The dermal microenvironment into which arthropod-borne pathogens are transmitted is acutely influenced by the pharmacological and immunomodulatory effects of vector saliva. Added to this is the anti-saliva immune response of hosts with previous exposure to uninfected bites. There is now substantial evidence that this unique context, bypassed by needle-injection models, can significantly influence the infectivity of arthropod-transmitted pathogens. For example, in naïve animals the infectivity of both the sandfly-borne parasite *Leishmania* and the tick-borne bacterium *Borrelia* is enhanced by the immunomodulatory properties of the vector's saliva, but in sensitized animals the delayed-type hypersensitivity reaction at the bite site and acquired immune response to saliva are detrimental to pathogenesis [17], [18]. In the case of West Nile virus infection, uninfected *Culex tarsalis* mosquitoes feeding on the footpads of mice followed immediately by needle injection of virus resulted in higher viremia at 24 and 48 h pi compared to needle infection alone [24]. This enhancement of infection was the same in mice presensitized to *Culex tarsalis* saliva.

Unlike other vector-pathogen systems, repeated prior exposure of mice to uninfected flea bites had no significant effect on transmission, mortality, or time to disease after challenge with *Y. pestis*-infected fleas compared to naïve controls (Fig. 7, Table 3). Injection of *Y. pestis*, either associated with flea bites or not, stimulated innate cell recruitment at 3 h which subsided at later time points despite concurrent bacterial replication. This is consistent with several studies showing that *Y. pestis* inhibits the inflammatory response until late in the disease progression [31], [68]–[70]. In addition, injection of *Y. pestis* in association with flea bites did not enhance or inhibit bacterial replication or dissemination, in keeping with a previous study which reported no difference in bubonic plague pathogenesis following id injection of BALB/c mice with or without the presence of *X. cheopis* SGE in the inoculum [71]. In summary, by all parameters tested here, previous exposure to flea bites had no effect on *Y. pestis* infection in mice, and the inflammation observed in naïve mice exposed to fleas was inhibited in the presence of *Y. pestis*. The generally non-stimulatory nature of flea bites, the host tolerance to them, and the anti-inflammatory faculties of *Y. pestis* likely explain why exposure history to flea saliva did not affect plague transmission and pathogenesis.

## Supporting Information

Figure S1 **Histological changes in skin of flea-exposed Balb/c mice 3 days after flea bites.** Representative examples of skin showing minimal inflammation (**A**, score = 1), and mild inflammation (**B**, score = 2); (**C**), unbitten control skin (score = 0). 2/5 and 3/5 ear samples had a score of 1 or 2, respectively. Mice were exposed to 20 fleas once per week for 10 weeks. Mean number of total flea bites per mouse ± s.d. = 101±21. Scale bars = 50 um.
(TIF)

Table S1 **Serum antibody responses to salivary gland extract in a group of 5 Balb/c mice exposed to 20 fleas 1×/week for 10 weeks.**
(DOCX)
