# Supplementary figures and images for: Evaluation of the Murine Immune Response to Xenopsylla cheopis Flea Saliva and Its Effect on Transmission of Yersinia pestis
Source: PLoS Negl Trop Dis. 2014 Sep 25;8(9):e3196. doi: 10.1371/journal.pntd.0003196 (PMC4177749; doi:10.1371/journal.pntd.0003196)

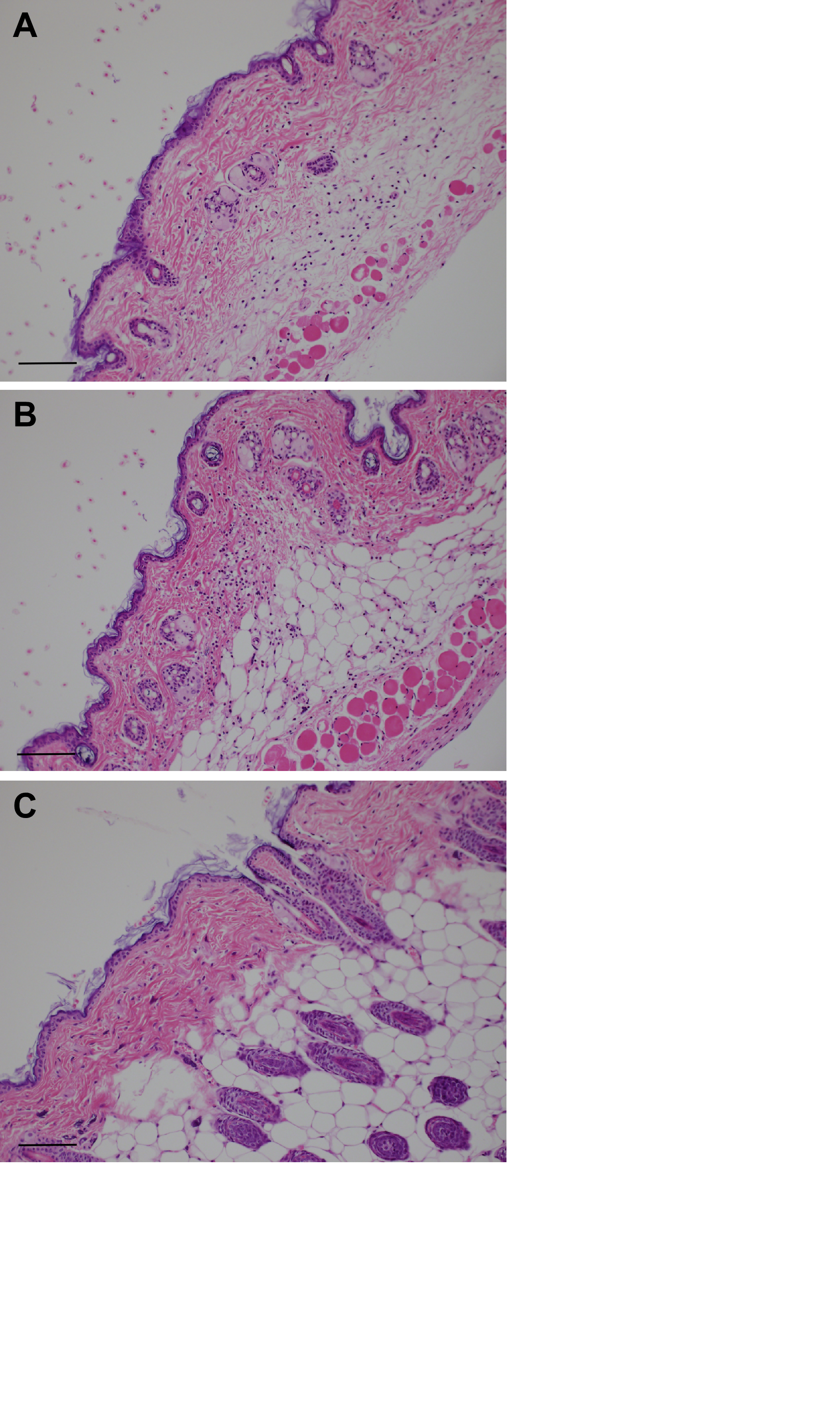

Supplement: Figure S1 — Histological changes in skin of flea-exposed Balb/c mice 3 days after flea bites. Representative examples of skin showing minimal inflammation (A, score = 1), and mild inflammation (B, score = 2); (C), unbitten control skin (score = 0). 2/5 and 3/5 ear samples had a score of 1 or 2, respectively. Mice were exposed to 20 fleas once per week for 10 weeks. Mean number of total flea bites per mouse ± s.d. = 101±21. Scale bars = 50 um. (TIF) [file pntd.0003196.s001.tif]
