# Supplementary material for: Evaluation of the Murine Immune Response to Xenopsylla cheopis Flea Saliva and Its Effect on Transmission of Yersinia pestis
Source: PLoS Negl Trop Dis. 2014 Sep 25;8(9):e3196. doi: 10.1371/journal.pntd.0003196 (PMC4177749; doi:10.1371/journal.pntd.0003196)
Supplement: Table S1 — Serum antibody responses to salivary gland extract in a group of 5 Balb/c mice exposed to 20 fleas 1×/week for 10 weeks. (DOCX) [file pntd.0003196.s002.docx]

**Table S1.** Serum antibody responses to salivary gland extract in a group

of 5 Balb/c mice exposed to 20 fleas 1x/week for 10 weeks.

|  |  | No. +^a^ | log_10_(U)^b^ |
| --- | --- | --- | --- |
| IgG | 5-week sera | 0/5 | 0 |
|  | 10-week sera | 5/5 | 1.94 ± 0.8 |
| IgM | 5-week sera | 2/5 | 0.84 ± 0.1 |
|  | 10-week sera | 1/5 | 0.90 |
| IgG1 | 10-week sera | 3/3 | 2.86 ± 0.3 |

^a^ELISA results; number positive out of total (No. +)

^b^Mean units of antibody (log_10_ (U) ± s.d.) of the positive samples. Positive

samples defined as having a mean log_10_ (U) > 2 s.d. above negative control sera.
